# Predicting the complicated neutropenic fever in the emergency department

**DOI:** 10.1136/emj.2008.064865

**Published:** 2009-10-15

**Authors:** J M Moon, B J Chun

**Affiliations:** Department of Emergency Medicine, Chonnam National University Hospital, Gwangju, South Korea

## Abstract

**Objectives:**

The purpose of this study was to identify independent factors that can be used to predict whether febrile neutropenic patients who appear healthy at presentation will develop subsequent complications, using variables that are readily available in the emergency department (ED).

**Method:**

The medical records of 192 episodes in which the patients presented to the ED with neutropenic fever resulting from chemotherapy, with an alert mental state and haemodynamic stability were retrospectively reviewed. Endpoints examined were fever response to administered antibiotics, death or severe medical complications during hospitalisation.

**Results:**

Thirty-eight episodes of neutropenic fever with complicated outcomes were identified from among a total of 192 episodes. Three parameters emerged as independent factors for the prediction of neutropenic fever with complications in the multivariate regression analysis: platelet count (130−450 × 10^3^ cells/mm^3^) <50 000 cells/mm^3^, serum C-reactive protein (CRP, 0.1–1 mg/dl) >10 mg/dl and pulmonary infiltration on chest *x* ray.

**Conclusions:**

Platelet count, CRP and pulmonary infiltration on chest *x* ray at presentation could be used to identify febrile neutropenic patients who will develop complications, and these factors may be useful in making treatment-related decisions in the ED.

Large studies have shown that neutropenic fever after chemotherapy causes death in 4–30% of patients and serious medical complications in 21% of patients.^1^ ^2^ However, it has been suggested that patients with febrile neutropenia are a heterogenous group in terms of medical complications and mortality. Recent advances in the treatment of neutropenic fever have highlighted the value of risk stratification and recommended outpatient treatment for low-risk patients.^1^ ^2^ ^3^ ^4^ ^5^ ^6^

The degree and duration of neutropenia have been identified as key factors related to the risk and outcome of infection.^4^ However, this finding was of little use to the treating physician because these factors can only be determined retrospectively.

Risk prediction models, including the Talcott model and the Multinational Association of Supportive Care of Cancer risk index scoring system, were developed.^1^ ^3^ However, the Talcott model requires information, such as tumour response to chemotherapy, which is not readily available in the emergency department (ED).^1^ It is possible that the patient’s medical records may not be available in the ED because the patient may be new to the institution. The Multinational Association of Supportive Care of Cancer risk index scoring system includes variables that may be graded differently by different physicians, such as tumour burden of illness.

Previous studies identified clinical or laboratory parameters that could serve as independent predictive factors for risk assessment, including age, acute leukaemia and white blood cell count.^7^ ^8^ ^9^ However, there is a slight limitation in that these factors were not selected as predictive factors in the other studies for the following reasons. First, the conclusions drawn from studies in children cannot be applied to adults as a result of the differences in comorbidity and the dose of chemotherapy administered. Second, the endpoints used in previous studies, such as bacteraemia, serious bacterial infection or clinical complications, were various.

Studies were recently conducted to analyse the usefulness of serum inflammatory markers, such as procalcitonin, IL-6 and C-reactive protein (CRP), in the assessment of the risk of febrile neutropenia.^10^ ^11^ ^12^ ^13^ However, these studies were primarily concerned with determining whether these inflammatory markers can identify bacteraemia rather than subsequent complications, and the value of the markers in predicting patient outcomes is limited.^10^ ^11^ ^12^ ^13^

Physicians will regard patients with neutropenic fever combined with shock at presentation as being in a critical condition and treat them intensively. It is thus important to avoid undertreating patients with complicated neutropenic fever, even though they may have appeared healthy upon presentation at the ED.

However, to our knowledge, no study has been conducted to identify the objective independent variables that can be easily assessed in the ED and use them to predict which patients with febrile neutropenia and haemodynamic stability at presentation will develop subsequent potentially serious complications.

Therefore, we sought to identify simple independent factors that can predict patients who develop subsequent complications after chemotherapy-induced neutropenic fever.

## Methods

### Design

The present study was a retrospective cohort study that was conducted using chart review methodology. This study was approved by our hospital’s institutional review board.

### Setting and population

This study was conducted at a single academic tertiary care centre with an annual ED census of 40 000 patients with or without cancer per year. The hospital is a large urban medical centre and is designated as one of five specialised cancer centres in South Korea.

We used the hospital electronic medical record system to obtain the medical record numbers of the patients enrolled in this study. The patient selection criteria were as follows: patients presenting to the ED with C00–C97 codes (malignant neoplasm) classified according to the International Classification of Diseases, version 10 between March 2004 and December 2007, neutrophil count less than 500 cells/mm^3^, age older than 18 years and systolic blood pressure greater than 90 mm Hg at presentation. Fever was defined as an oral temperature greater than 38.3°C on a single occasion or greater than 38.0°C for one hour or longer during the first 24 h after admission.^14^ A total of 254 episodes was identified. However, six episodes presenting to the ED in an altered mental state were excluded. In addition, we excluded nine episodes who underwent radiotherapy before or during the febrile neutropenic episode, 27 episodes who were transferred to other hospitals or our hospital in the middle of treatment for neutropenic fever, 11 episodes who presented with neutropenic fever as an initial clinical manifestation of unevaluated underlying cancer, seven episodes with incomplete data and two episodes with AIDS.

Finally, 192 febrile neutropenic episodes occurring in 168 patients were analysed. Each febrile neutropenic episode was considered to be a new event.

### Protocol

The first author assessed whether the patient met the defined criteria for combined fever and chemotherapy-related neutropenia. The first assistant, who was unaware of the purpose of the study, classified each patient as with or without risk of complications during hospitalisation. Later, a second author, who was unaware of the risk classification and had previous experience with data abstraction from electronic medical records, collected the variables such as initial laboratory findings, empirically administered antibiotics and duration of hospitalisation. To test the accuracy of the risk assessment by the first assistant, a second assistant, who was not listed as an author, evaluated 15% of the patient medical records selected by random sampling. There was no disagreement on the risk assignment.

Complicated neutropenic fever was defined when the fever did not resolve within 5 days of starting treatment, in case of death, or in patients with serious medical complications that needed treatment and occurred during hospitalisation.

### Measurement

The following data were collected: (1) variables that were readily available at presentation without information about the medical history of the patient and tested for their predictive value, such as age, sex, tumour type, laboratory variables and the interpretation of chest *x* ray by a radiologist; (2) other variables related to patients, such as latency to the development of neutropenic fever after recent chemotherapy, previous medical history, prophylactic administration of antibiotics or antifungal agents and presumed site of infection at presentation; (3) variables related to treatment and outcome, including empirically administered antibiotics, microbiological test results, duration of neutropenia and fever, complications, duration of hospitalisation and survival.

The patients were divided according to microbiologically defined infection with or without bacteraemia, clinically defined infection and unexplained fever according to previously published definitions.^15^

### Data analysis

Baseline data on characteristics and outcomes were summarised by frequency tabulation or mean values. Univariate analysis of the association between each covariate and outcome was performed using the χ^2^ test. Continuous variables tested in the univariate analyses were categorised on the basis of clinical arguments according to physician’s judgement or previously published reports. All covariates with p values less than 0.05 were considered sufficient for the inclusion of a variable in multivariate stepwise logistic regression analysis, with a significance level of p<0.05. Estimated odds ratios and confidence intervals were calculated for all significant variables. All reported p values are two-sided, and all statistical analyses were performed using SPSS version 15.0.

## Results

### Patient characteristics

A total of 154 episodes without complications and 38 episodes with serious complications was analysed. Table 1 shows the characteristics and demographic data of the 192 episodes.

**Table 1:** Clinical characteristics of the 192 episodes of febrile neutropenia

Clinical characteristic	Episode
Mean age (years)	53.5 (SD 12.4)
Gender (male/female)	70/122
Underlying cancer, N (%)	
Breast cancer	65 (33.9%)
Haematological cancer	59 (30.7%)
Pulmonary cancer	24 (12.5%)
Gastrointestinal cancer	23 (12.0%)
Skeletal cancer	6 (3.1%)
Hepatobiliary cancer	3 (1.6%)
Other solid tumour	12 (6.3%)
Comorbidity, N (%)	
Recent surgery (<6 months)	46 (24.0%)
Previous neutropenic fever	47 (24.5%)
Diabetes mellitus	19 (9.9%)
Heart failure	5 (2.6%)
Prophylactic medication, N (%)	
Antibiotic medication	24 (12.5)
Antifungal medication	6 (3.1%)
Central venous catheter in place, N (%)	11 (5.7%)
First installed chemotherapy, N (%)	61 (31.8%)
Latency after chemotherapy <7 days, N (%)	41 (24%)
Duration of fever at presentation <24 h, N (%)	135 (70.3%)
Presumed site of infection at presentation, N (%)	
Gastrointestinal tract	65 (33.9%)
Unexplained fever	62 (32.3%)
Pulmonary tract	44 (22.9%)
Other	21 (10.9%)

The mean age in the study group was 53 years. The mean blood pressure was 126.2 mm Hg (SD 16.8) and the temperature at presentation was 37.9°C (SD 1.0). Fifty-nine episodes had haematological malignancies, of which lymphoma (47 episodes) was the most common underlying haematological malignancy, followed by 11 cases of leukaemia and one case of multiple myeloma.

All patients were admitted and treated with empiric broad-spectrum antibiotics as inpatients. A single antibiotic (meropenema, imipenem, cefepime or ceftazidime) was administered to 23 episodes. A total of 169 episodes (88.0%) was treated with the above-mentioned antibiotics in combination with an aminoglycoside. Eighty-three episodes with neutrophil counts less than 100 cells/mm^3^ received haematopoietic growth factors during hospitalisation.

### Outcome

Nine (5.8%) of the episodes in the group of patients without complications and eight (21.1%) of the episodes in the group of patients with complications had a microbiological pathogen (p = 0.001). In addition, of the 60 episodes who had an unexplained fever, 57 were in the group of patients without complications (p = 0.001). However, there was no difference in the incidence of clinically defined infection between the groups with and without complications.

Neutropenia (2.6 days (SD 2.0) vs 3.8 days (SD 1.9), p = 0.001) lasted significantly longer in the group of patients with complications than in the group of patients without complications. Patients without complications were discharged 6.6 days (SD 4.1) after hospitalisation, whereas patients with complications were hospitalised for 18.2 days (SD 16.8; p<0.001).

The most frequent complications were hypotension (34.2%), followed by respiratory failure (23.7%; table 2).

**Table 2:** Serious medical complications developed in patients with complicated neutropenic fever

Complications	N (%)
Hypotension*	13 (34.2%)
Respiratory failure†	9 (23.7%)
Disseminated intravascular coagulation	7 (18.4%)
Renal failure‡	2 (5.3%)
Severe bleeding to require tranfusion	2 (5.3%)
Altered mental state	1 (2.6%)
Arrhythmia requiring treatment	1 (2.6%)
Others	3 (7.9%)

*Hypotension was defined as systolic blood pressure less than 90 mm Hg or the need for pressor to maintain blood pressure. †Respiratory failure was defined as arterial oxygen pressure less than 60 mm Hg while breathing room air or the need for mechanical ventilation. ‡Renal failure required treatment with fluids, dialysis or any other intervention.

These complications occurred within one to 9 days after admission. Other complications deemed clinically significant by the investigator included two cases of electrolyte disturbance and one case of hepatic failure. The fever did not resolve within 5 days after starting treatment in 14 episodes, and seven of them did not show any kind of serious medical complication. Seven (3.6%) of the patients died, and each of these patients had at least two serious medical complications.

### Risk stratification

Univariate analysis of the clinical parameters readily available in the ED was performed (table 3).

**Table 3:** Univariate analysis of outcomes for patients with neutropenic fever

	Total	Without	With	p Value
	N (192)	complications	complications	
		N (154)	N (38)	
Age >65 years	37 (19.3%)	28 (18.2%)	9 (23.7%)	0.441
Male	70 (36.5%)	49 (31.8%)	21 (55.3%)	0.007
Haematological cancer	59 (30.7%)	41 (26.6%)	18 (47.4%)	0.013
Laboratory findings				
WBC <500 cells/mm^3^	83 (43.2%)	60 (39.0%)	23 (60.5%)	0.016
Platelets <50 000 cells/mm^3^	46 (24.0%)	26 (16.9%)	20 (52.6%)	<0.001
Monocytes <100 cells/mm^3^	92 (47.9%)	66 (42.9%)	26 (68.4%)	0.005
Neutrophils <100 cells/mm^3^	115 (59.9%)	91 (59.1%)	24 (63.2%)	0.647
Lymphocytes <100 cells/mm^3^	37 (19.3%)	25 (16.2%)	12 (31.6%)	0.032
Total protein (6–8.3 g/dl) <6.0 g/dl	51 (26.6%)	36 (23.4%)	15 (39.5%)	0.044
Albumin (3.5–5 g/dl) <3.0 g/dl	25 (13.0%)	17 (11.0%)	8 (21.1%)	0.100
BUN (8–20 mg/dl) >20 mg/dl	32 (16.7%)	22 (14.3%)	10 (26.3%)	0.075
Creatinine (0.5–1.2 mg/dl) >1.2 mg/dl	19 (9.9%)	12 (7.8%)	7 (18.4%)	0.049
CRP (0.1–1.0 mg/dl) >10 mg/dl	78 (40.6%)	52 (33.8%)	26 (68.4%)	<0.001
Positive test for urine nitrates	18 (9.4%).8%)	16 (10.4%)	2 (5.3%)	0.332
Pulmonary infiltration	18 (9.4%)	3 (1.9%)	15 (39.5%)	<0.001

The normal ranges of complete blood counts are as follows: white blood cells (WBC) (4.8−10.8×10^3^ cells/mm^3^), platelets (130−450×10^3^ cells/mm^3^), 2–9% monocytes, 50–75% neutrophils and 20–40% lymphocytes of WBC. BUN, blood urea nitrogen.

In the multivariate analysis, platelets less than 50 000 cells/mm^3^, CRP greater than 10 mg/dl and pulmonary infiltration on chest *x* ray were determined to be independent factors for the prediction of neutropenic fever with complications that requires maximal attention from physicians (table 4).

**Table 4:** Independent predictors identified by multivariate analysis

Variable	β	OR	p Value	95% CI
CRP >10 mg/dl	1.000	2.719	0.028	1.111 to 6.651
Platelets <50 000 cells/mm^3^)	1.606	4.982	0.001	2.000 to 12.408
Pulmonary infiltration	3.407	30.167	<0.001	7.281 to 124.998

CRP, C-reactive protein; OR, odds ratio.

## Discussion

The number of patients who appear healthy at presentation but will develop serious complications and in whom independent variables can predict such an outcome in this group is an important question for emergency medicine physicians. Therefore, we only enrolled patients who were alert and had systolic blood pressure greater than 90 mm Hg at presentation. In addition, we observed the elevation of body temperature during the first 24 h after presentation, in order to include patients who had taken an antipyretic agent before presentation and did not have a body temperature greater than 38.3°C at presentation.

In the present study, 19.8% of episodes experienced at least one serious medical complication or episode of prolonged fever. Early predictive markers of neutropenic febrile patients who are likely to develop serious complications are thus needed to allow the rapid evaluation and treatment of febrile neutropenia, as in 19.8% of the episodes in our study.

In our study, we excluded parameters that were affected by the subjective judgement of the physician and those that were obtained retrospectively or through detailed analysis of the patient’s medical history, even though these factors were considered to be predictive factors in previous reports. Finally, platelet count, CRP and pulmonary infiltration were shown to have a significant association with outcome. The clear advantage of these significant variables is that there is no observer variability and they can be very readily assessed on presentation.

The initial complete blood cell count had been investigated in several studies of risk assessment. Studies in the field of paediatrics by Rackoff *et al*,^7^ Baorto *et al*^16^ and Klaassen *et al*^17^ proposed that the monocyte count with a specific cutoff value was a significant factor in the development of bacteraemia or severe bacterial infection. In a recent prospective study,^12^ none of the haematological parameters were found to be independent variables for the prediction of complications in adults with chemotherapy-induced febrile neutropenia. In our study, only platelets less than 50 000 cells/mm^3^ was predictive of a complicated neutropenic fever. It may be possible that the platelet count acts as a surrogate marker of the host response to inflammation and exposure to activated coagulation factors to accelerate disseminated intravascular coagulopathy before the initiation of therapy.

The level of serum CRP was also recognised as an independent factor in the multivariate model. CRP was sensitive for the prediction of neutropenic fever with shock or complex infection in haematological malignancies.^18^ It is possible that the production of CRP is stimulated by cytokines in response to infection or inflammation. However, CRP failed to predict deterioration in adults with neutropenic fever subsequent to chemotherapy.^11^ ^12^ These controversial consequences might result from its late response to inflammation and a wide range of serum levels related to the amount of tissue injury.

It is noteworthy that abnormality on chest *x* ray was significantly associated with outcome, as in several previous studies.^3^ ^8^ In particular, it was noted in 15–25% of all episodes of febrile neutropenia after chemotherapy for haematological malignancies, and they could be responsible for the mortality rates of up to 23–50%.^19^ ^20^ In our study, pulmonary infiltration had a higher odds ratio than other significant variables.

We suggest that the initial platelet count and serum CRP as well as pulmonary infiltration could be used to identify patients with neutropenic fever who appear well at admission but will develop complications. This may maximise the chance of identifying patients who are most likely to benefit from rapid admission and intensive treatment. We did not insist that the patients without predictive factors should not be admitted. We cautiously suggested outpatient treatment in these cases after observing the patient for 2–24 h to confirm the assessment of risk, assess the patient’s medication tolerance and provide patient education, if treatment guidelines in an individual institution are established.

This study has several limitations. First, this study was performed at a single institution, which is one of five specialised cancer centres in our country. Therefore, our findings may not be representative of all other institutions, and they may vary according to the chemotherapy regimen. Second, the results of our study are based on a retrospective analysis of a small population, and should therefore be interpreted with some caution and confirmed through prospective studies. However, there were no missing data regarding the laboratory findings that were not influenced by reviewer bias and inconsistencies in patient medical records. Third, there was no standard guideline for the administration of empirical antimicrobial agents due to the retrospective nature of the study, and the physician’s familiarity affected the choice of antimicrobial agent. However, in our study, extended antimicrobial therapy was administered initially and then modified based on microbiological findings and the patient’s clinical response.^21^ Fourth, haematopoietic growth factor was administered to 83 patients in our study. However, there was no difference in the administration of haematopoietic growth factor between the two groups, and the benefits of haematopoietic growth factor have not been definitively proved.^22^
